# A review of *Madagopsina* Feijen, Feijen & Feijen (Diptera, Diopsidae) with description of a new species, key to the species, and discussion of intrageneric relationships

**DOI:** 10.3897/zookeys.1057.67433

**Published:** 2021-08-24

**Authors:** Hans R. Feijen, Frida A. A. Feijen, Cobi Feijen, Benoît Gilles

**Affiliations:** 1 Naturalis Biodiversity Center, P. O. Box 9517, 2300 RA Leiden, Netherlands Naturalis Biodiversity Center Leiden Netherlands; 2 ETH Zürich, Institute of Integrative Biology (IBZ), 8092 Zürich, Switzerland ETH Zürich, Institute of Integrative Biology Zürich Switzerland; 3 Passion-Entomologie Association, 327 rue de Périgueux, 16000 Angoulême, France Passion-Entomologie Association Angoulême France

**Keywords:** Allometry, catalogue, Madagascar, stalk-eyed flies, wing morphometry

## Abstract

For the recently established genus *Madagopsina* (Diopsidae, stalk-eyed flies), *Madagopsinamakayensis* Feijen, Feijen & Feijen, **sp. nov.** is described from Madagascar. A concise catalogue is given for the genus and an identification key is presented for its six species. The differential character states are listed for the two species groups of the genus: the *Madagopsinaapollo* species group and the *Madagopsinaapographica* species group. The intrageneric relations are discussed based on morphology, geometric morphometrics analysis of wing shape, and allometric data for eye span against body length. Each of these three procedures places the new species in the *M.apollo* species group with *Madagopsinaparvapollina* as its closest relative. New records are presented for *M.apographica* and *M.parvapollina*.

## Introduction

In 2018, Feijen et al. erected *Madagopsina* Feijen, Feijen & Feijen and *Gracilopsina* Feijen, Feijen & Feijen as endemic genera for Madagascar. These new genera were placed in a Diopsidae clade with irrorated wings, named the *Teleopsis* genus group. In *Madagopsina*, two earlier described species were placed: *Diopsisapollo* Brunetti and Diopsis (Eurydiopsis) apographica Séguy. In addition, three new species were described for *Madagopsina*: *M.freidbergi*, *M.parvapollina*, and *M.tschirnhausi*. Shortly after the 2018 publication, a single specimen of *Madagopsina* was received which turned out to be an undescribed species. This species is described herein. In *Madagopsina*, two species groups were distinguished by Feijen et al. (2018), the *Madagopsinaapollo* species group and the *Madagopsinaapographica* species group. Based on morphology, allometric data and geometric morphometrics analysis of wing shape, the new species is placed in the *M.apollo* species group. Because of the description of the new species, the sets of character states for the two species groups need to be adapted. A concise catalogue for *Madagopsina* is presented, as well as a new identification key to the six species of the genus. Some new *Madagopsina* records are included in the catalogue. The first live photographs of *M.parvapollina* are presented as these high-resolution pictures nicely show differential characters for the species group. In Feijen and Feijen (2021 in press), a key to the Afrotropical genera of Diopsidae is presented along with a synopsis of the Afrotropical Diopsidae fauna, including the genus *Madagopsina*.

## Materials and methods

The description of *M.makayensis* Feijen, Feijen & Feijen, sp. nov. is based on a single male specimen that was preserved in alcohol. The holotype is now pinned with the genitalia placed in a genitalia tube attached to the pin. Some additional records for *Madagopsina* became known via photographs placed on www.iNaturalist.org. For the rate of dimorphism *D*, the difference between males and females in allometric slope for eye span on body length is used in the Diopsidae (Baker and Wilkinson 2001). Details on procedures for preparing genitalia slides, and procedures for taking measurements are given in Feijen et al. (2018). For information on morphological terminology and on photographic equipment used, the reader is referred to the same source. Some changes have been made to the terminology used: the aedeagus is now referred to as phallus, while the apodeme of the surstylus is now called the apophysis. The procedures for the wing geometric morphometrics analysis are described in Feijen et al. (2018). The following institutional codens and abbreviations are used:

**RMNH**Naturalis Biodiversity Center (formerly Rijksmuseum van Natuurlijke Historie), Leiden, The Netherlands,

**AU** Approximately Unbiased *p*-value,

**BP** Bootstrap Probability values,

**D** Rate of Dimorphism,

**SE** Standard Error.

## Taxonomy

### Family Diopsidae

Taxon classificationAnimaliaDipteraDiopsidae

Billberg, 1820

FA3303AA-7F0D-53D5-A7F8-E69F7BE58CB0

Diopsidae Billberg, 1820: 115 (as Natio Diopsides). Type genus: Diopsis Linnaeus, 1775: 5.

### Genus Madagopsina

Taxon classificationAnimaliaDipteraDiopsidae

Feijen, Feijen & Feijen, 2018

4D04C4C8-5A6C-5BB2-BD78-10C9E10C267F

Figures 1–2 , 3–4 , 5–8 , 9–11 , 12–14 , 15–17 , 18–22 , 23–28 , 29 , 30 , 31

Madagopsina Feijen, Feijen & Feijen, 2018:145. Type species Diopsisapollo Brunetti, 1928.
Eurydiopsis sensu Séguy & Vanschuytbroeck (nec Frey) - in part; Shillito 1971: 287; Feijen 1981: 482; Feijen 1989: 63; Feijen and Feijen 2013: 182, 185.

#### Remarks.

A concise catalogue for the genus is given below. For details on the type series, records, and combinations to various other genera of the earlier described species can be referred to Feijen et al. (2018). Reference is now made to new records which appeared after this publication. The new species *Madagopsinamakayensis* Feijen, Feijen & Feijen, sp. nov. is added.

### Madagopsina apographica

Taxon classificationAnimaliaDipteraDiopsidae

(Séguy, 1949)

2F88E1A7-404F-5F9F-A3BF-BD079CFF0099

Figures 12 , 26

Diopsis (Eurydiopsis) apographicus Séguy, 1949: 69.
Eurydiopsis anjahanaribei Vanschuytbroeck, 1965: 336.
Madagopsina apographica ; Feijen et al. 2018: 151.

#### New records.

Madagascar, 1 ♀, Fianarantsoa, Vatovavy, Fitovinany, Ifanadiana, 21°15'34"S, 47°24'55"E, 977 m, 7.xi.2014, lemurtaquin, (ref. www.inaturalist.org/observations/36199753); 1 ?sex (probably ♀), Antsiranana, Sava, Sambava, rainforest, 14°26'60"S, 49°43'10"E, 1310 m, 30.x.2016, Éric Mathieu (ref. www.inaturalist.org/observations/69807405). The new records fall well within the eastern forests distribution as indicated in Feijen et al. (2018).

### Madagopsina apollo

Taxon classificationAnimaliaDipteraDiopsidae

(Brunetti, 1928)

DD9ACBC3-1C0B-5A0F-93CA-0CF36EDBA883

Figures 9 , 23

Diopsis apollo Brunetti, 1928: 280.
Madagopsina apollo ; Feijen et al. 2018: 160.

### Madagopsina freidbergi

Taxon classificationAnimaliaDipteraDiopsidae

Feijen, Feijen & Feijen, 2018

FC96DACB-0EFD-576A-BF5D-70A83954A4E7

Figures 13 , 27

Madagopsina freidbergi Feijen et al., 2018: 165.

### Madagopsina parvapollina

Taxon classificationAnimaliaDipteraDiopsidae

Feijen, Feijen & Feijen, 2018

54566C25-0A0A-5E4B-8564-2EC56B31AD2A

**Figures 1–2.**
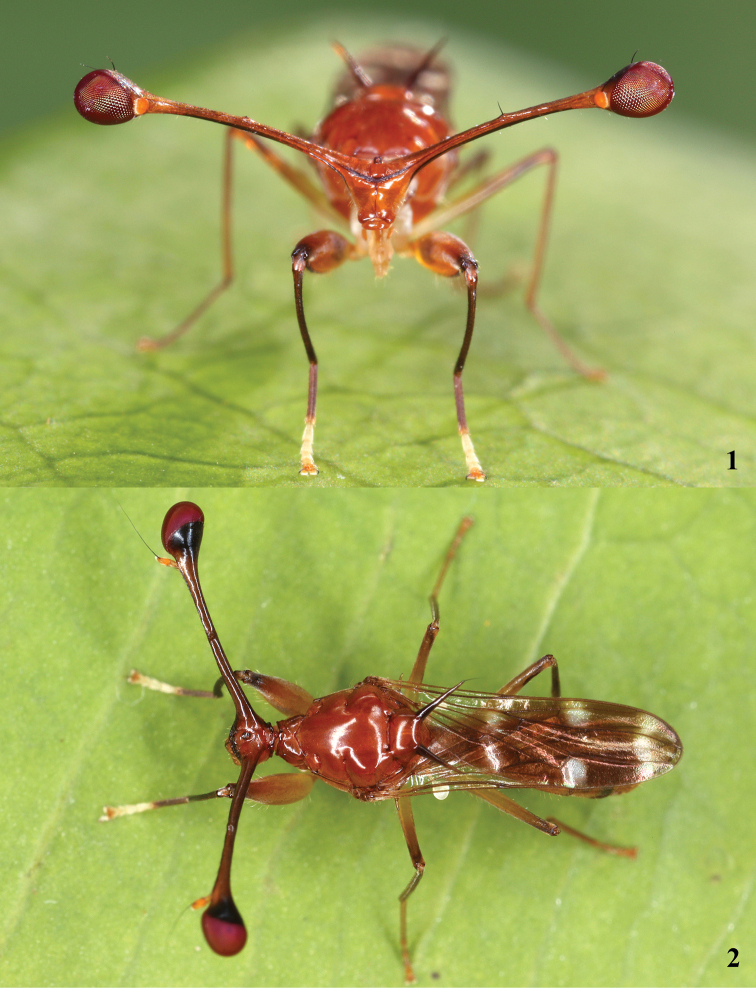
*Madagopsinaparvapollina*, live photographs by Gernot Kunz, Mahajanga, Boeny (www.inaturalist.org/observations/20766277) **1** anterior view **2** dorsal view.

Figures 1 , 2 , 11 , 25

Madagopsina parvapollina Feijen et al. 2018: 172.

#### New records.

Madagascar, 1 ♂, Mahajanga, Boeny, 16°24'44"S, 45°18'48"E, 123 m, 23.x.2016, Gernot Kunz (ref. www.inaturalist.org/observations/20766277); 1 ?sex (probably ♂), Mahajanga, Boeny, Soalala, 16°26'4"S, 45°21'20"E, 138 m, Josiane Lips, Olivier Testa (ref. www.inaturalist.org/observations/37503778 and www.inaturalist.org/observations/37503777), the photograph formed part of a batch made during a caving expedition in Namoroka caves, while all pictures were taken in caves or at the entrance; 1 ♂ Makay, canyon, sous-bois, rive d’une rivière [undergrowth, riverbank], 21°10'11"S, 45°22'15"E, 528 m, 30.vii–3.viii.2017, leg. Benoît Gilles. The new records fall well within the western forests distribution as indicated in Feijen et al. (2018).

### Madagopsina tschirnhausi

Taxon classificationAnimaliaDipteraDiopsidae

Feijen, Feijen & Feijen, 2018

F2C83F4E-A48C-5210-B906-612CB8431FCB

Figures 14 , 28

Madagopsina tschirnhausi Feijen et al. 2018: 178.

### Madagopsina makayensis

Taxon classificationAnimaliaDipteraDiopsidae

Feijen, Feijen & Feijen sp. nov.

C83F35AD-89DD-54BE-89B0-677EE4666AA7

http://zoobank.org/A6766D1D-FCB3-49B6-A640-278DDE98BB4A

Figs 3–4 , 5–8 , 10 , 15–17 , 18–22 , 24 , 29 , 30 , 31

#### Type material.

***Holotype*,** ♂ (RMNH), Madagascar, Makay, canyon, sous-bois, rive d’une rivière [undergrowth, riverbank], 21°10'11"S, 45°22'15"E, 528 m, 30.vii–3.viii.2017, leg. Benoît Gilles.

#### Diagnosis.

*Madagopsinamakayensis* Feijen, Feijen & Feijen, sp. nov. can be recognised by its medium size (body length ♂ 7.3 mm), brown colour (however, due to conservation in alcohol it is likely that all the brown colours would be more yellowish in a live specimen, like in the other *Madagopsina* species), body mainly thinly pruinose (pollinose) with few small setulae, only katepisternum and katepimeron glossy, absence of facial teeth, medium-sized inner vertical seta (1.7 × stalk diameter), scutellar spines 2.0 × as long as scutellum, quite large apical seta (45% of scutellar spine length), incrassate fore femora with around 48 tubercles, irrorated wings with three vague crossbands including an H-shaped configuration with central and preapical crossbands, wing apex infuscated, central band slightly broader than preapical band, pale wing spots in cell r2+3 and cell m1, a vague pale spot in cell m4, abdomen club-shaped, no pruinose spots on tergites, ♂ spiracles 7 in slit of synsternite 7+8, surstyli rounded and bulbous with an apically rounded apophysis, microtrichia on posterior apical third, phallapodeme with ratio posterior arm/anterior arm 1.05, straight ejaculatory apodeme with only a slight sickle-shape apically, phallus remarkably broad and sclerotised, assumed moderate sexual dimorphism with regards to eye span (*D* ≈ 1.0), ratio eye span/body length ~ 1.20 in ♂. *Madagopsinamakayensis* Feijen, Feijen & Feijen, sp. nov. belongs to the *M.apollo* species group, which furthermore includes *M.apollo* and *M.parvapollina*.

**Figures 3–4.**
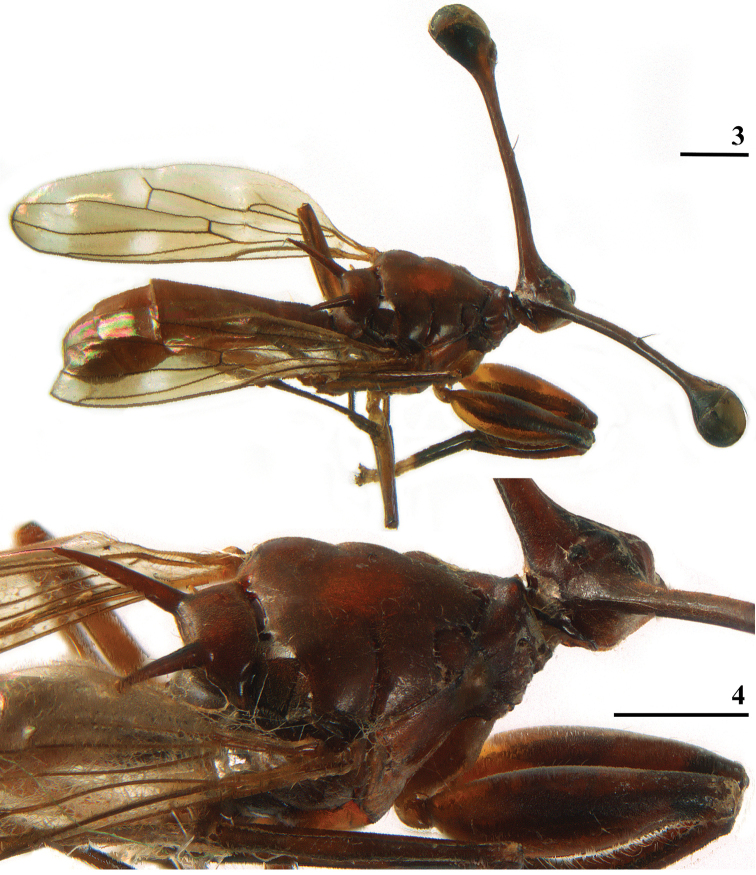
*Madagopsinamakayensis* Feijen, Feijen & Feijen, sp. nov., ♂, holotype, Makay **3** habitus, dorsolateral view **4** thorax, dorsolateral view. Scale bars: 1 mm.

#### Description.

***Measurements***. Body length ♂ 7.3 mm; eye span 8.8 mm; wing length 5.9 mm; length of scutellar spine 1.01 mm.

**Figures 5–8.**
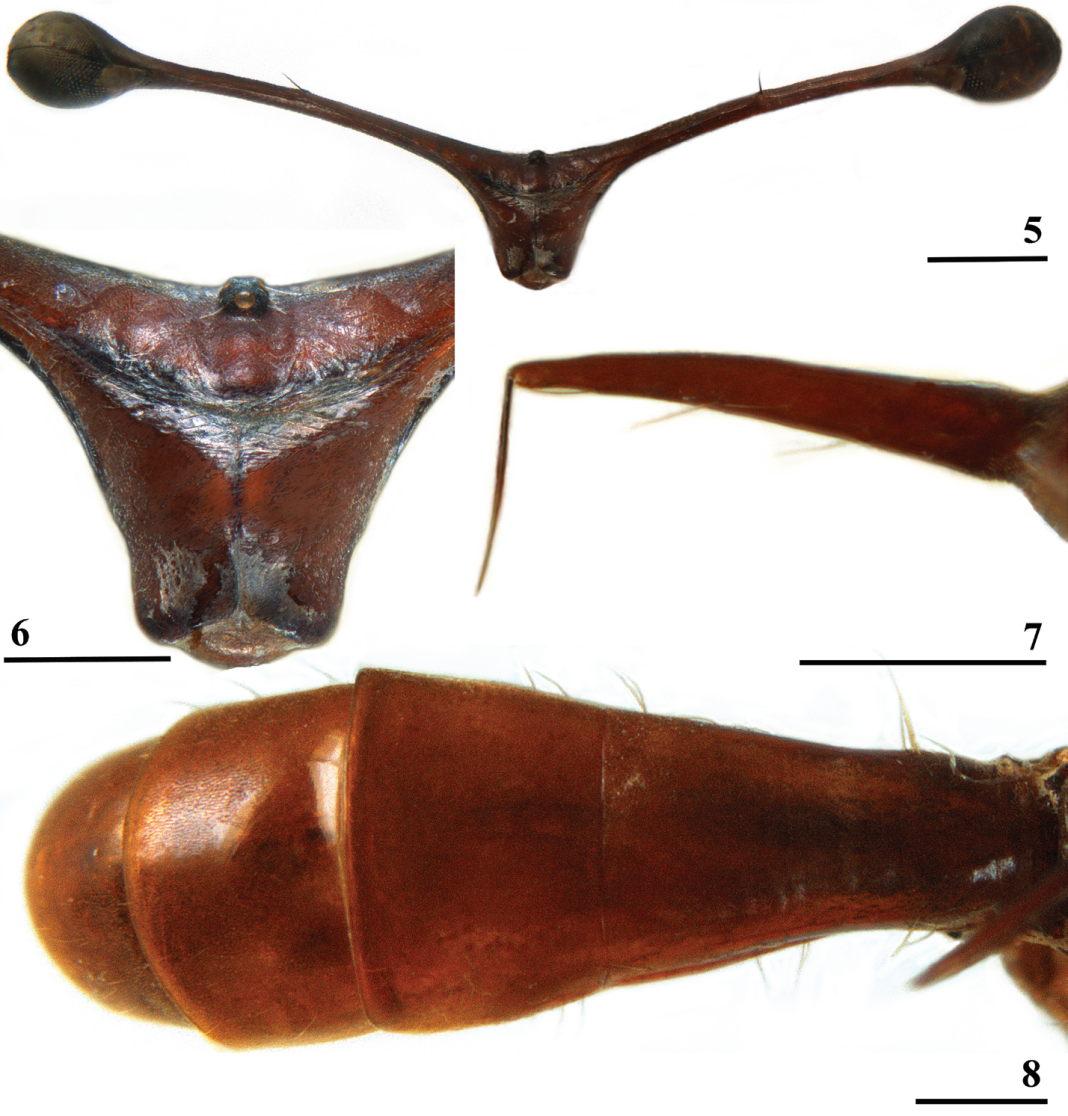
*Madagopsinamakayensis* Feijen, Feijen & Feijen, sp. nov., ♂, holotype, Makay **5** head, anterior view **6** central head, anterior view **7** scutellar spine and apical seta, inner view (seta not in natural position in line with spine) **8** abdomen, dorsal view. Scale bars: 1 mm (**5**); 0.5 mm (**6–8**).

***Head*.** Central part brown, ocellar tubercle and arcuate groove dark brown; central head thinly pruinose (Figs 3–6); an elongate bulbous medial ridge in front of ocellar tubercle, parallel grooves on both sides of this ridge, lateral areas of frons flat; medial occiput flat; face convex in profile, facial corners square, no facial teeth (Figs 5, 6); clypeus small, not protruding; arista finely microtrichose on less than basal half; the rate of dimorphism cannot be calculated, but in the graph (Fig. 31) with the allometric lines for the three species of the *M.apollo* species group it can be seen that the single data point is located in line with the allometric line for *M.parvapollina* males, while given that the slopes for the females for the species must be almost identical, it follows that *D* for the new species must be almost identical to the D = 0.98 for *M.parvapollina* or slightly higher (see also the section “Allometric aspects with regard to eye span” below); eye span large in male (119.6% of body length), also a comparison of this ratio eye span/body length of the single male with the mean ratio eye span/body length of the other *Madagopsina* species (Feijen et al. 2018) supports the view that this is a dimorphic species with a moderate rate of dimorphism *D* ≈ 1.0; stalks thinly pruinose, brown, broad apical parts dark, funiculus brown, pruinose; inner vertical seta medium-sized, 1.7 × diameter of eye stalk (Figs 3, 5), base of inner vertical seta a minor elevation, one-eighth diameter of the stalk; outer vertical seta broken off; central head and stalks with a few tiny white setulae.

***Thorax*.** Collar, scutum, scutellum and postscutellum pruinose, brown (Figs 3, 4), spines glossy; pleura dorsally brownish pruinose, katepisternum and katepimeron largely glossy; ratio scutal length/scutal width ~ 0.80; scutellar spines almost straight, diverging under an angle of ~ 65°, ratio scutellar spine/scutellum in ♂, 2.00, ratio scutellar spine/body length in ♂, 0.14; metapleural spines well developed, pointing almost laterally (Fig. 3); apical seta quite large, 45% of length of scutellar spine, posteriorly directed (Figs 4, 7, in Fig. 7 the seta is not in its natural, posteriorly directed, position); scutum almost devoid of setulae, scutellar spines with each ~ 10 small setulae, not on warts.

***Wing*.** Irrorated with a rather vague, brownish, H-shaped configuration; apex (8% of wing length) with brownish infuscation (convex on proximal side); 3 crossbands, the basal and central band hardly separated, a pale preapical band and three pale spots (Figs 3, 10); preapical crossband (distal leg of H) broad, marginally darker than other bands and with slightly irregular edges; preapical band connected to central band in cell r1, in cell r4+5 and around veins R2+3 and R4+5; central band slightly broader than preapical band and with ill-defined proximal edge, only in cell m4 vaguely separated from basal band; basal band running from cell c to posterior wing margin, widening posteriorly; this infuscation pattern creates a pale (but not hyaline) preapical band between dark preapical band and infuscated wing apex, two pale spots between central and dark preapical bands (one in cell r2+3 and one in cell m1), and one vague pale spot centrally in cell m4 between basal and central band (Figs 3, 10); glabrous basal areas include basal apices of cell c and cell r1, basal half of cell br, basal quarter of cell bm+dm except for posterior margin and basal third of cell cua; vein M4 reaching to just beyond halfway the wing margin.

**Figures 9–11.**
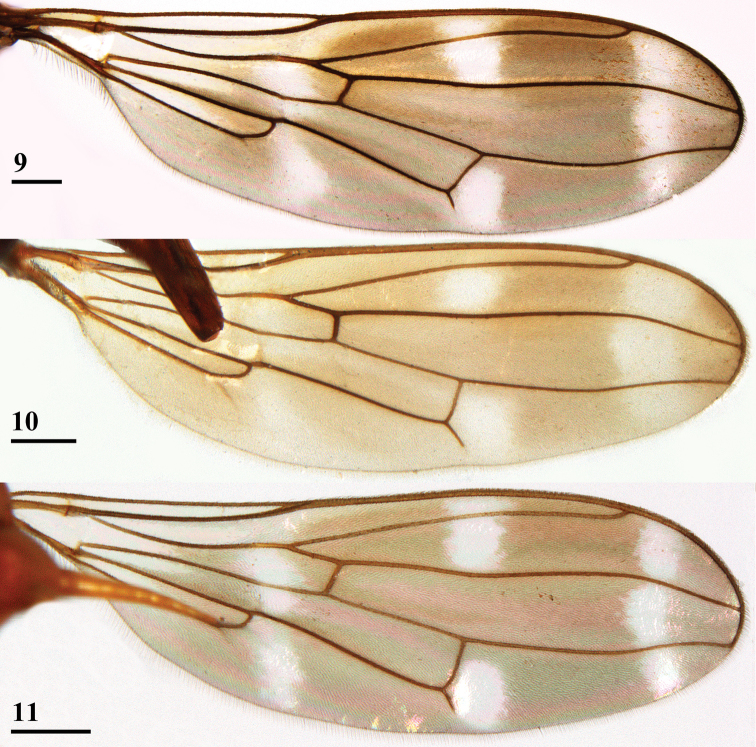
*Madagopsinaapollo* species group, dorsal view of wings **9***M.apollo*, ♂, Ambohitra **10***M.makayensis* Feijen, Feijen & Feijen, sp. nov., ♂, holotype, Makay **11***M.parvapollina*, ♀, paratype, Ankarana. Scale bars: 0.5 mm. Figures **9, 11** (Feijen et al. 2018, figures 6, 7).

***Legs*.** Coxa 1 pale yellowish, glossy but with dense white pruinescence on anterior side, trochanter 1 pale, pruinose; fore femur yellowish brown but dorsally darker, glossy but dorsally and apically pruinose; fore tibia and metatarsus darker brown, other fore tarsi pale and covered with whitish pruinescence; mid- and hind legs more uniformly yellowish, femora pruinose dorsally and with dark brown spot on apical fifth; femur 1 (Figs 3, 4) incrassate in ♂ (ratio of length/width 3.2), two rows of tubercles on distal two-thirds, inner row in ♂ with 25 and 28 tubercles (mean 26.5, *n* = 2), outer row in ♂ with 21 and 22 tubercles (mean 21.5, *n* = 2); femur 1 with whitish setulae ventrally.

**Figures 12–14.**
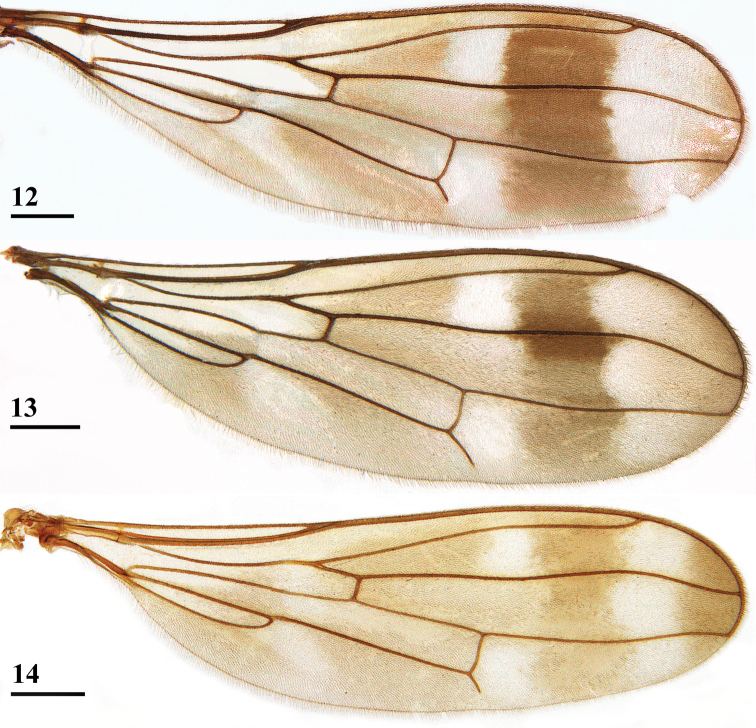
*Madagopsinaapographica* species group, dorsal view of wings **12***M.apographica*, ♀, Fianarantsoa **13***M.freidbergi*, ♀, paratype, Vohimana **14***M.tschirnhausi*, ♂, holotype, Mount Ambre. Scale bars 0.5 mm. Figures **12–14** (Feijen et al. 2018, figures 8–10).

**Figures 15–17.**
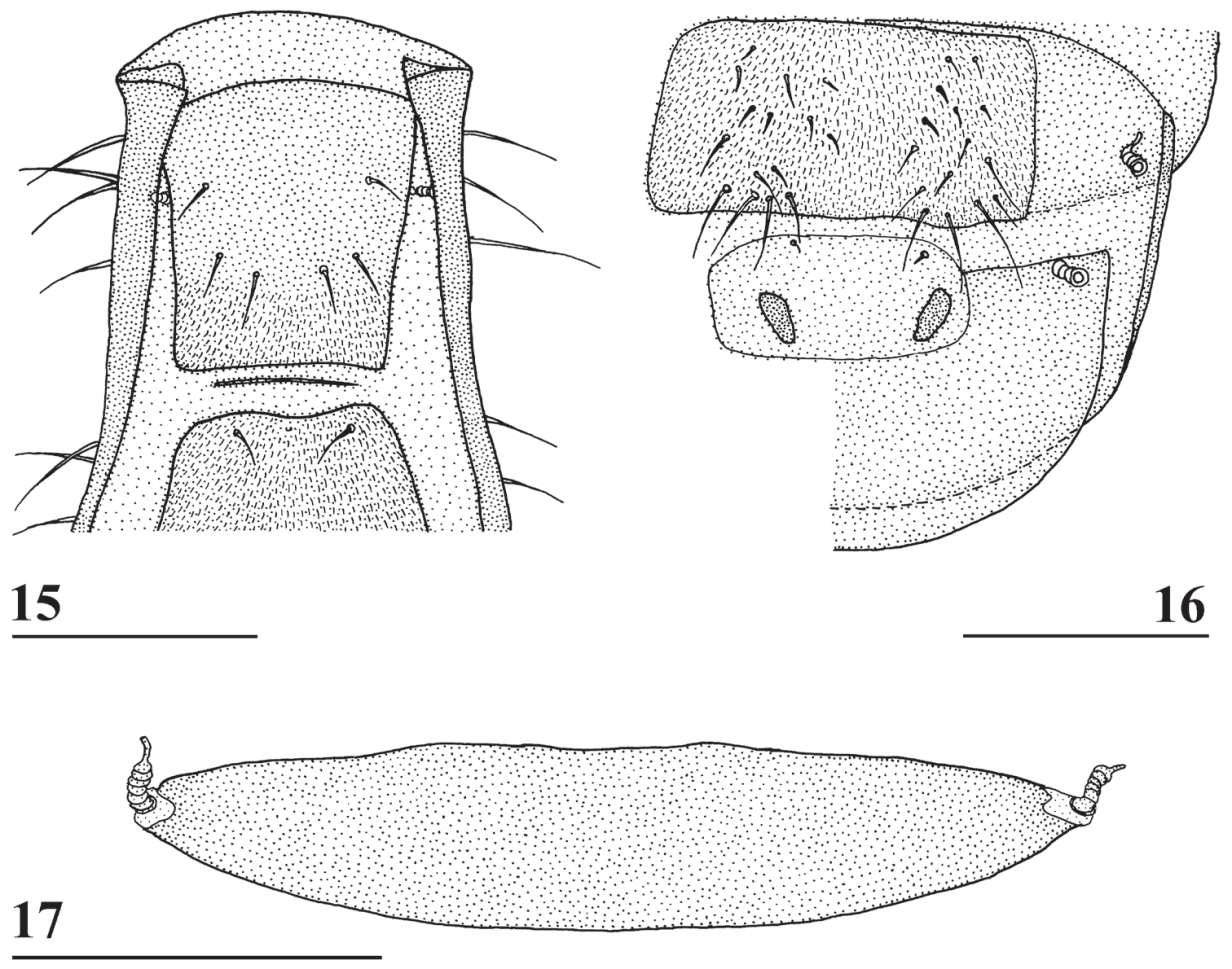
*Madagopsinamakayensis* Feijen, Feijen & Feijen, sp. nov., ♂, holotype, Makay **15** basal section of abdomen with intersternite 1–2, ventral view **16** sternites 5 and 6, ventral view **17** synsternite 7+8, ventral view. Scale bars: 0.5 mm.

***Preabdomen*.** Abdomen club-shaped (ratio length/broadest width 2.8); syntergite gradually widening posteriorly, seam between tergites 1 and 2 not visible, suture between tergites 2 and 3 distinct (Fig. 8); tergites uniformly yellowish brown (Figs 3, 8), thinly pruinose; syntergite basally with white setulae laterally, otherwise tergites with a few whitish setulae; anterior line-like section (intersternite 1–2) of sternite 2 not linked to main sternite 2 (Fig. 15); ratio length sternites 1+2+3/width posterior sternite 2 2.8 in ♂, ratio length/width of sternite 2 1.2 in ♂; sternites very pale, pruinose (except for basal two-thirds of sternite 1); spiracle 1 in tergite (Fig. 15).

**Figures 18–22.**
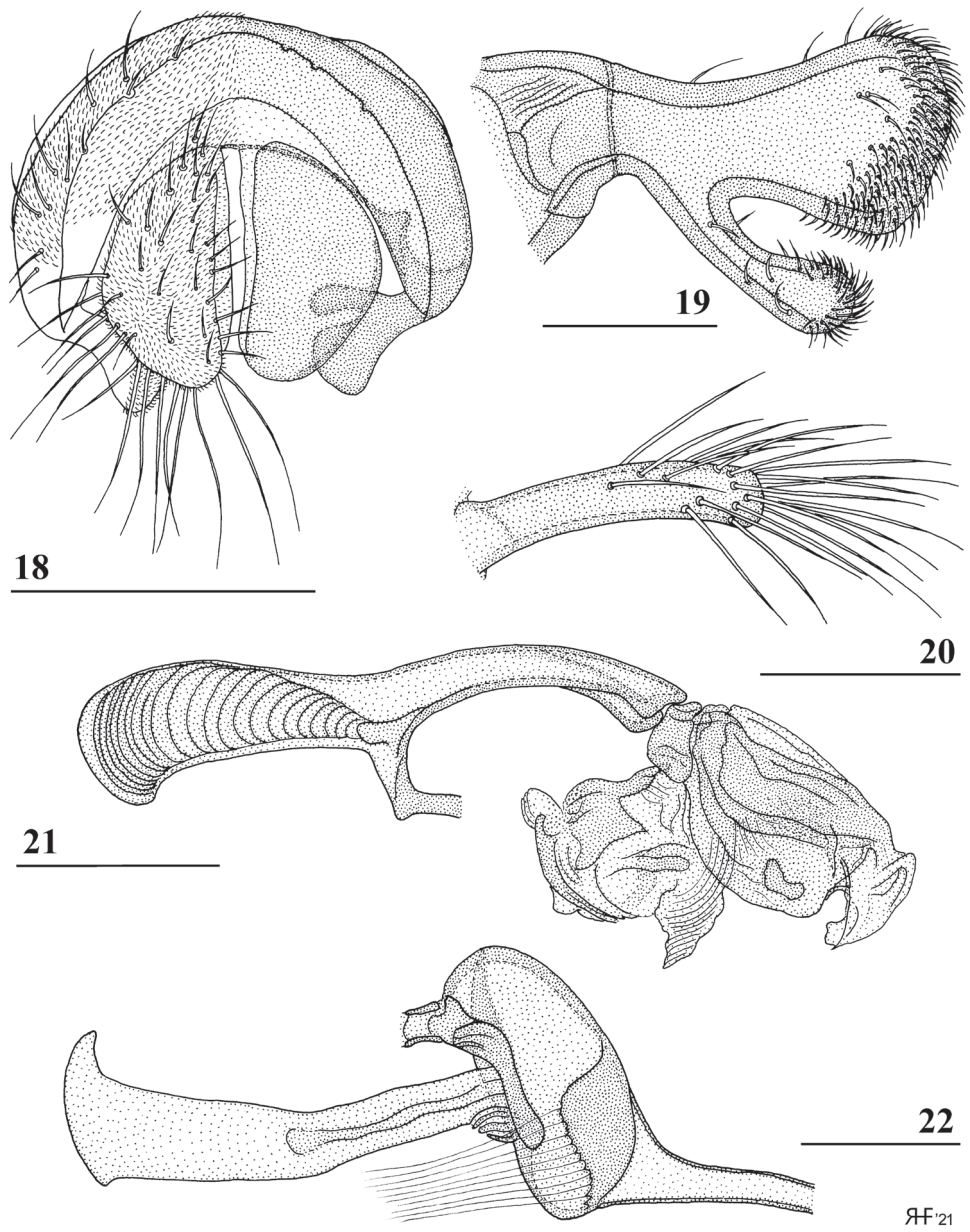
*Madagopsinamakayensis* Feijen, Feijen & Feijen, sp. nov., ♂, holotype, Makay **18** epandrium with surstyli and cerci, posterior view **19** surstylus, inner view **20** hypandrial clasper, lateral view **21** phallapodeme and phallus, lateral view **22** ejaculatory apodeme and sac. Scale bars: 0.5 mm (**18, 21**); 0.1 mm (**19, 20, 22**).

***Postabdomen male*.** Sternite 4 a rectangular plate; sternite 5 (Fig. 16) a rectangular plate, slightly more sclerotised laterally; sternite 6 vague with a pair of small sclerotised sections (Fig. 16); synsternite 7+8 quite large, symmetrical, narrowing laterally, lateral slits enclosing hardly sclerotised areas (Fig. 17); both spiracles located in the lateral slits of the synsternite (Fig. 17); epandrium (Fig. 18) rounded, with ~ 11 pairs of setulae, ventrally bare, otherwise clothed in microtrichia; surstyli (Figs 19, 24) articulated, apically broadening, apex rounded and bulbous, with a long, apically broadening and rounded, apophysis; in posterior view (Fig. 24) a few small setulae on apical halves of surstylus and apophysis with the apical third of the surstylus and only the apex of the apophysis clothed in microtrichia, on inner side only microtrichia on the apices of surstylus and its apophysis with a few small setulae on apophysis and apical third of surstylus (Fig. 19); surstyli interconnected via thin, rod-like processus longi (Fig. 18); cerci rather broad, ratio of length/width 1.9, basally and apically tapering, apex rounded, clothed in microtrichia and a set of setulae, some of the apical setulae almost as long as the cerci (Fig. 18); hypandrial clasper (Fig. 20) straight and rod-like with relatively long setulae on distal half; phallapodeme solidly built and rather straight (Fig. 21), anterior arm rounded apically, posterior arm slightly longer than anterior arm (ratio posterior arm/anterior arm 1.05) and strongly bifurcated to accommodate the very broad phallus; phallus (Fig. 21) a rather short complex of lobes and sclerites, remarkably broad and heavily sclerotised, intromittent organ very short; ejaculatory apodeme straight, hardly broadening apically except for a small sickle-shape of apex (Fig. 22), ejaculatory sac rounded.

#### Distribution and habitat.

The new species is only known from the Makay massif in Toliara province. The Makay is a mountain range of almost 4000 km^2^ in southwestern Madagascar. The altitude varies from 200 m at the bottom of canyons to 1000 m for the plateaus. The Makay with its exceptional biodiversity (see Wendenbaum 2011) is considered to be one of the least studied areas in Madagascar. Its forests belong to the deciduous, seasonally dry, western forests of low altitude (Du Puy and Moat 1999). In the dry season, wet areas remain near the rivers. Many Diopsidae, including aggregations, were observed on vegetation in wet, shady places. The single specimen of this new species was collected in undergrowth along a riverbank at an altitude of 527 m. On the same location the following Diopsidae were collected: 5 ♀ and 5 ♂ *Sphyracephalabeccarii* (Rondani), 5 ♀ and 2 ♂ *Diopsisnigrosicus* Séguy, and 1 ♂ *M.parvapollina*.

#### Etymology.

This species is named *M.makayensis* Feijen, Feijen & Feijen, sp. nov., referring to the place of origin of the holotype.

##### Key to the species of *Madagopsina*

This key is a revised version of the Madagopsina section of the key in Feijen et al. (2018). It now also includes *M.makayensis* Feijen, Feijen & Feijen, sp. nov. for which only the male is known. The couplet separating the two species groups has been changed to accommodate the new species. In the 2018 key also an error occurred: in the couplet separating *M.apollo* and *M.parvapollina*, the character states for the apical seta should have been reversed.

**Figures 23–28.**
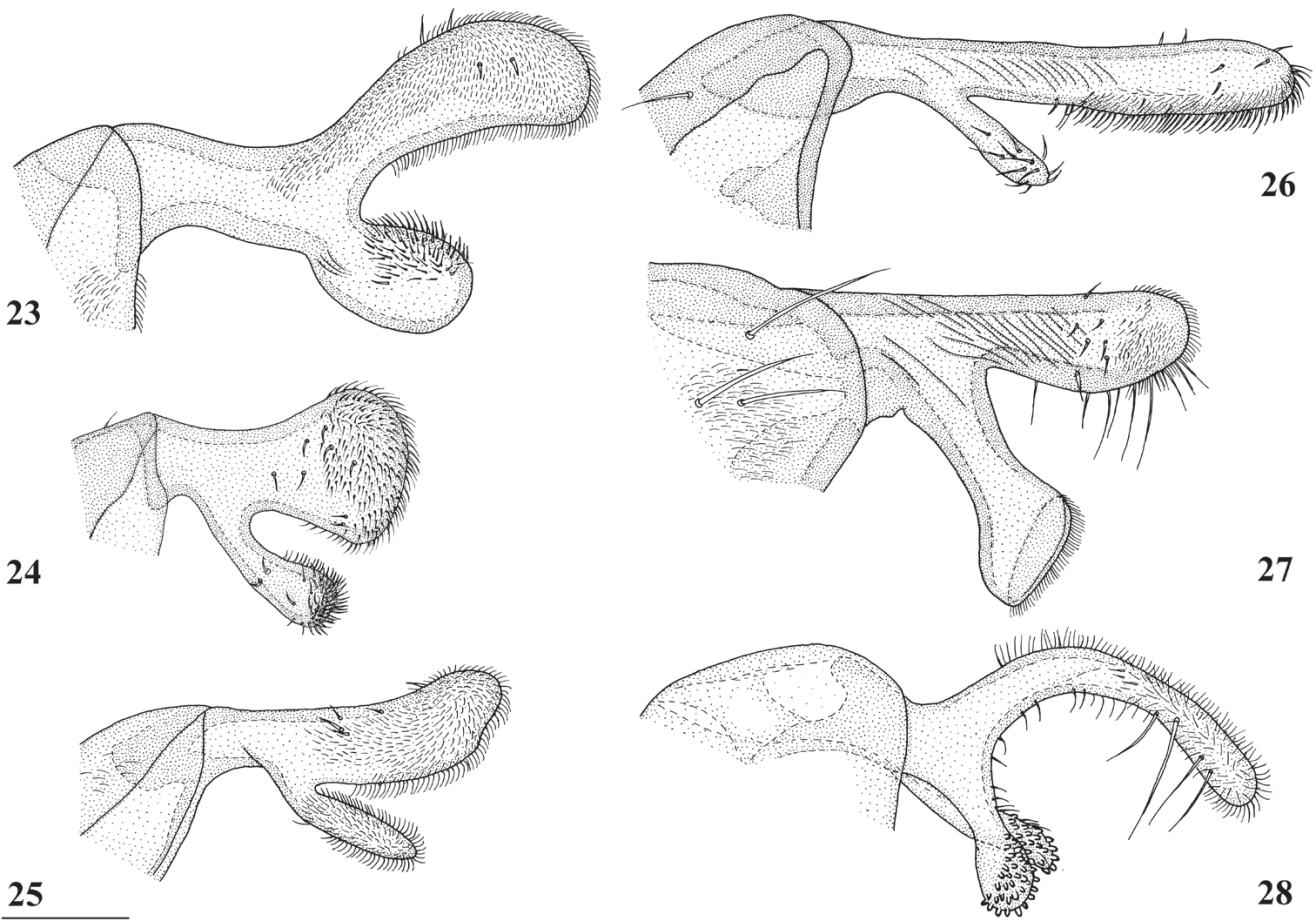
Posterior view of *Madagopsina* surstyli **23–25***M.apollo* species group **26–28***M.apographica* species group **23***M.apollo***24***M.makayensis* Feijen, Feijen & Feijen, sp. nov. **25***M.parvapollina***26***M.apographica***27***M.freidbergi***28***M.tschirnhausi*. Scale bar 0.1 mm (all drawn to the same scale). Figures **25–28** (Feijen et al. 2018, figures 187, 184, 186, 188).

**Table:** 

1	Fore femur incrassate in females (ratio length/width 3.4–3.5) and males (ratio length/width 3.2–3.7) (Figs 2, 4), pleurotergal spines laterally directed (Fig. 2), dark preapical wing band (width 18–20% of wing length) as broad as central band and equal in colour (Figs 9–11), abdomen club-shaped (ratio length/broadest width ≤ 3) (Fig. 8), tergites glossy, ratio length sternites 1+2+3/width posterior sternite 2 2.8–3.1, posterior arm of phallapodeme longer than anterior arm (ratio ~ 1.05–1.40) (Fig. 21)	**2 (*Madagopsinaapollo* species group)**
–	Fore femur moderately incrassate to slender in females (ratio length/width 4.6–6.0) and males (ratio length/width 4.6–6.3), pleurotergal spines posterolaterally directed, dark preapical wing band (width 13–14% of wing length) distinctly narrower than central band and darker (Figs 12–14), abdomen slender (ratio length/broadest width ~ 4), tergites thinly pollinose with a pair of pollinose lateral spots on tergite 3, ratio length sternites 1+2+3/width posterior sternite 2 4.1–4.8, posterior arm of phallapodeme shorter than anterior arm (ratio ~ 0.71–0.93)	**4 (*Madagopsinaapographica* species group)**
2	Large – females on average 9.6 mm (range 8.3–10.2), males on average 9.5 mm (range 8.0–10.1), inner vertical seta 0.8 × stalk diameter, small apical seta (23% of length of scutellar spine), male sternite 5 without combs, apophysis of surstylus short (~ 30% of length of surstylus) and bulbous (Fig. 23), subanal plate heart-shaped with bulbous lateral areas	*** Madagopsina apollo ***
–	Medium-sized – females on average 6.1 mm (range 5.4–6.8), males on average 6.3 mm (range 5.6–7.0) or just larger to 7.3 mm (*M.makayensis* Feijen, Feijen & Feijen, sp. nov.), inner vertical seta 1.6–1.7 × stalk diameter (Figs 1, 3), medium-sized apical seta (37–43% of length of scutellar spine) (Fig. 2, 7), male sternite 5 with or without posterior combs of spine-like setulae, apophysis of surstylus long (> 55% of length of surstylus) and slender (Figs 24, 25), subanal plate triangular (not yet known for *M.makayensis* Feijen, Feijen & Feijen, sp. nov.)	**3**
3	Body length of male 7.3 mm, apical seta 45% of length of scutellar spine, anterior central hyaline wing spot in cell r2+3 and not extending into cell r1 (Fig. 10), fore femora with ~ 48 tubercles, male sternite 5 without combs, surstylus and its apophysis both apically broadening and rounded (Fig. 24)	***Madagopsinamakayensis* Feijen, Feijen & Feijen, sp. nov.**
–	Body length males on average 6.3 mm (range 5.6–7.0), apical seta on average 37% of length of scutellar spine, anterior central hyaline wing spot in cells r1 and r2+3 (Fig. 11), fore femora with ~ 36 tubercles, male sternite 5 with posterior combs of spine-like setulae, surstylus and its apophysis both straight, slender and not apically broadening (Fig. 25)	*** Madagopsina parvapollina ***
4	Inner vertical seta 2.3 × stalk diameter, femur 1 moderately incrassate in females and males (ratio length/width in both sexes 4.6), ratio scutellar spine/scutellum 2.1–2.3, dark preapical wing band rather vague but slightly darker than central band (Fig. 14), basal wing band extending through cell br (Fig. 14), cell br with microtrichia on apical half, tergite 3 with a pair of tiny posterolateral pollinose spots, surstylus strongly curved (Fig. 28)	*** Madagopsina tschirnhausi ***
–	Inner vertical seta 1.1–1.4 × stalk diameter, femur 1 slender in females (ratio length/width 5.3–5.9) and males (ratio length/width 5.3–6.3), ratio scutellar spine/scutellum 2.5–3.1, dark preapical wing band distinct and much darker than central band (Figs 12, 13), basal wing band not extending anteriorly of vein M1 (Figs 12, 13), cell br with microtrichia only on apical 10%, tergite 3 with laterally a pair of large pollinose spots, surstylus straight (Figs 26, 27)	**5**
5	Inner vertical seta 1.4 × stalk diameter, femur 1 slender in females and males (ratio length/width 5.3), ratio scutellar spine/scutellum 2.9–3.1, preapical dark band uniformly dark, only paler in cell r1 (Fig. 12), pollinose spots on tergite 3 posterolaterally located, ♀ sternite 8 divided in two sclerites, apophysis of surstylus less than half the size of central surstylus (Fig. 26)	*** Madagopsina apographica ***
–	Inner vertical seta 1.1× diameter of stalk, femur 1 very slender in females (ratio length/width 6.0) and males (ratio length/width 6.3), ratio scutellar spine/scutellum 2.5, preapical dark band with distinctly darker spot around vein R4+5 (Fig. 13), pollinose spots on tergite 3 mediolaterally located, ♀ sternite 8 a single sclerite, apophysis of surstylus equal in size to central surstylus (Fig. 27)	*** Madagopsina freidbergi ***

## Discussion

### Geometric morphometrics analysis of wing shape

**Figure 29.**
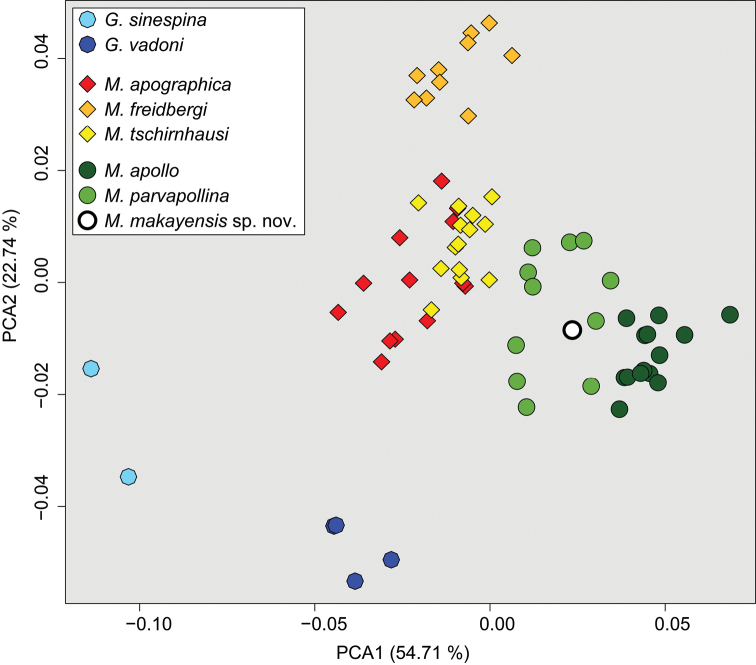
Principal component analysis of wing venation morphometry for the two species of *Gracilopsina* and six species of *Madagopsina*: biplot of the first two PCA axes.

**Figure 30.**
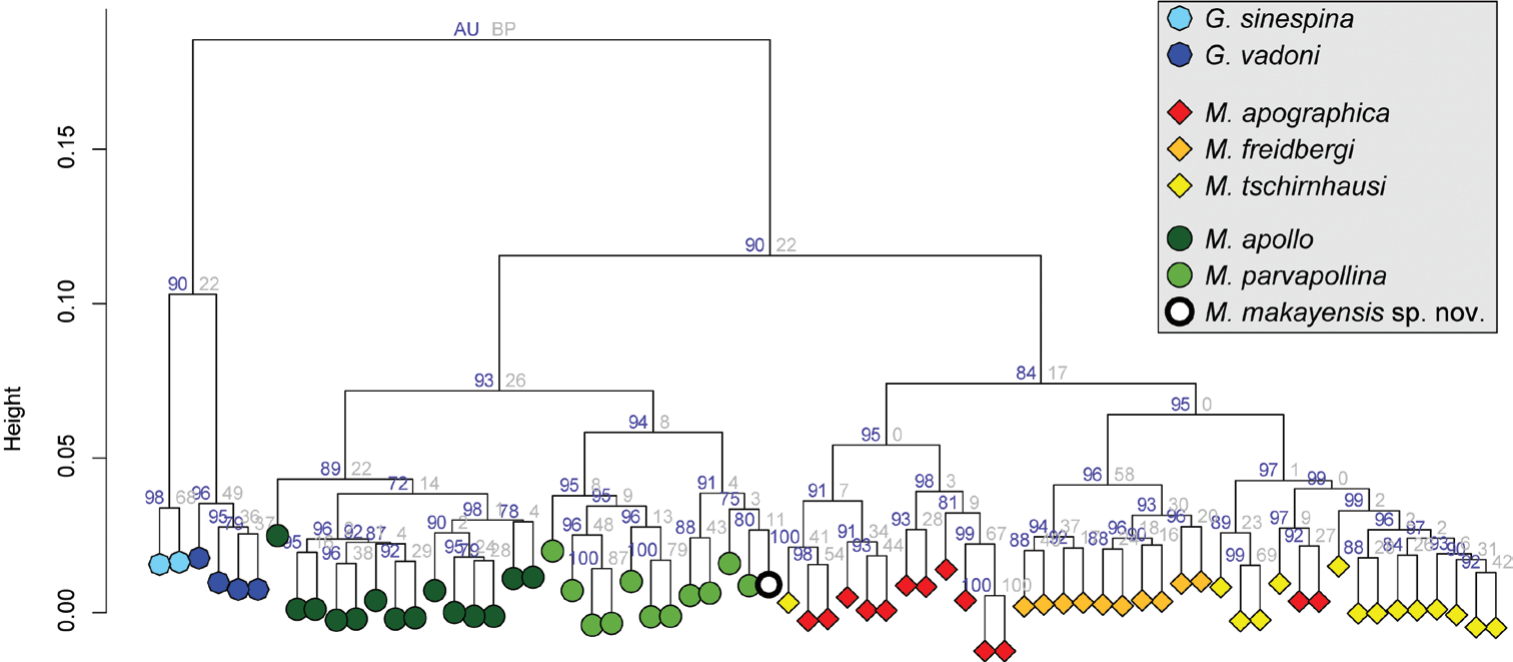
Cluster dendrogram for the Euclidian distance in wing morphometry PCA for the *Gracilopsina* and *Madagopsina* species using the complete clustering method. Branch labels give the approximately unbiased *p*-value (AU) and bootstrap probability (BP) values (%).

Feijen et al. (2018) proved that principal component analysis (PCA) of wing morphometry was powerful enough to recover the *Madagopsina* and *Gracilopsina* taxa previously delimited by adult morphology. Their biplot of the first two PCA axes showed clear distinction between the two genera and seven species while these two axes explained 54.8% and 22.9% of variation. Only specimens from *M.tschirnhausi* and *M.apographica* overlapped slightly. The *M.apographica* species group and the *M.apollo* species group were also delimited. Feijen et al. (2018) stated that the limited intraspecific variation in the PCA plot could, to some extent, be explained by the fact that the wings in the, mostly, pinned flies are often not perfectly flat. So, the PCA leads to a cluster pattern that is in accordance with morphological characters. The same pattern was seen in the hierarchical clustering analysis of PCA scores. Only three of 68 specimens were assigned to the wrong species cluster using the ‘complete’ cluster method. Both analyses were now repeated with inclusion of the wing data for the single specimen of the new species. The biplot of the first two PCA axes (Fig. 29) shows the distinction between the two genera and eight species, while the two axes explained 54.7% and 22.7% of variation. *M.makayensis* Feijen, Feijen & Feijen, sp. nov. is placed squarely in the cluster for the *M.apollo* species group, while within this group it is more closely related to *M.parvapollina* than to *M.apollo*. This same pattern is seen in the hierarchical clustering analysis of PCA scores using the complete cluster method (Fig. 30). This method showed that within *Madagopsina* the three species *M.apollo*, *M.parvapollina* and *M.makayensis* Feijen, Feijen & Feijen, sp. nov. form one cluster (the *M.apollo* species group, - AU = 93, BP = 26), while *M.freidbergi*, *M.tschirnhausi*, and *M.apographica* form a distinct second cluster (the *M.apographica* group – AU = 84, BP = 17). *M.makayensis* Feijen, Feijen & Feijen, sp. nov. is placed within the cluster for *M.parvapollina*.

### Allometric aspects with regard to eye span

**Figure 31.**
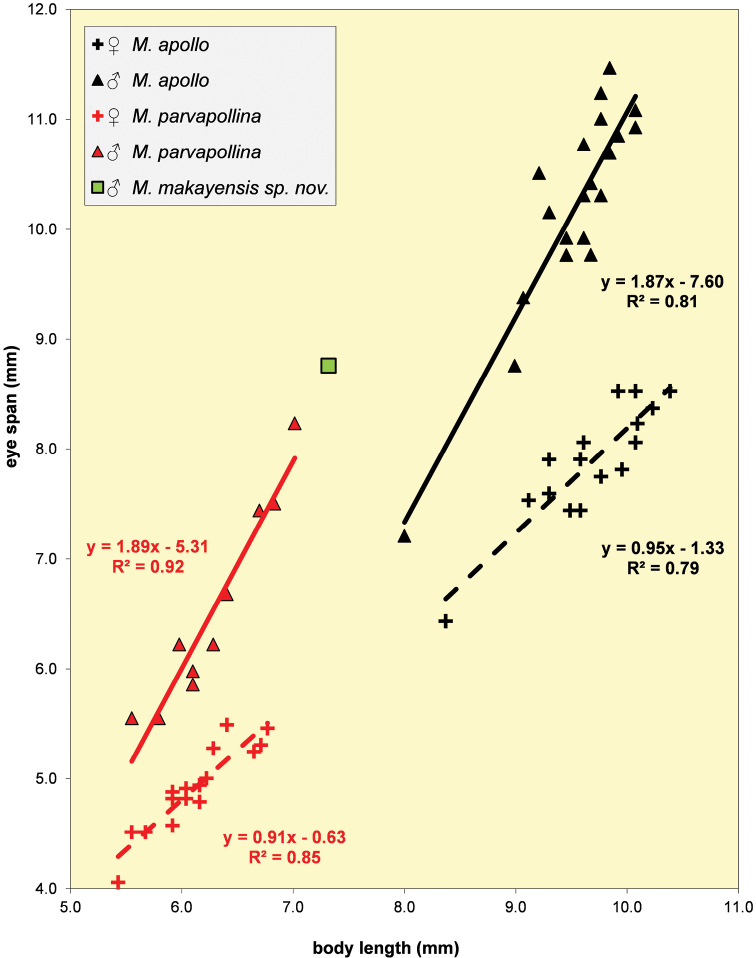
Eye span plotted against body length for the three species in the *Madagopsinaapollo* species group: *M.apollo*, *M.parvapollina* and *M.makayensis* Feijen, Feijen & Feijen, sp. nov. Note the position of the single data point for the ♂ of the latter species, in line with the ♂ data points for *M.parvapollina*.

For all five *Madagopsina* species included in Feijen et al. (2018), graphs were presented for eye span plotted against body length for both sexes. The differences in allometric slopes for males and females indicated the rate of sexual dimorphism *D* for the species. Between the species the allometric slopes for males varied from 1.64–2.13 and for females from 0.85–1.21. Of special interest were the allometric lines for the two species then forming the *M.apollo* species group: *M.apollo* and *M.parvapollina* (Fig. 31). According to Feijen et al. (2018) the two species are externally very similar. They can in the first place be distinguished by the well-separated size ranges (see also Fig. 31). Comparison of the allometric lines for the two species, showed that the female lines are collinear, but given the size difference well separated. In fact, the female lines for the *M.apographica* species group also do not differ much from those of the *M.apollo* group. The slopes of the male lines for *M.apollo* and *M.parvapollina* were almost similar with 1.87 and 1.89, respectively, but the intercepts are distinct. This leads to two parallel lines (Fig. 31). Now that *M.makayensis* Feijen, Feijen & Feijen, sp. nov. has become the third species in the *M.apollo* group, its place can be considered in the graph with the allometric lines for the other two species. Only one male specimen is available for *M.makayensis* Feijen, Feijen & Feijen, sp. nov., so a single data point is available in the graph (Fig. 31). The single male data point is in line with the allometric line for *M.parvapollina* males, which forms an indication that the new species has a closer relationship to *M.parvapollina*. The new species is probably also slightly larger than *M.parvapollina*. Although no females were available for *M.makayensis* Feijen, Feijen & Feijen, sp. nov. it can be assumed that the allometric line for these females will be collinear with those for *M.apollo* and *M.parvapollina*. Given also that *D* = 0.98 for *M.parvapollina*, it can safely be predicted that *M.makayensis* Feijen, Feijen & Feijen, sp. nov. is a clear dimorphic species with a low rate of dimorphism *D* ≈ 1.0. Another indication that *D* for the new species must be quite similar to those for the other two species rests on the similarity in the ratio eye span / body length. For the male *M.makayensis* Feijen, Feijen & Feijen, sp. nov. this comes to 1.20, while for males of *M.apollo* and *M.parvapollina* this ratio was 1.07 ± SE 0.01 and 1.04 ± SE 0.02, respectively.

### Male genitalia

According to Feijen et al. (2018), preliminary results from divergence dating analysis suggest a minimum age estimate of around 10 million years for the divergence of *M.freidbergi* and *M.tschirnhausi*. However, reaching convergence in divergence dating analysis proved difficult and longer runs will later be required. Feijen et al (2018) considered the large differences in postabdominal structures in *Madagopsina* as additional support that its species diverged long ago. In *M.makayensis* Feijen, Feijen & Feijen, sp. nov., the male genitalia are also quite distinct from the other *Madagopsina* species. The major differences in surstyli for the six species are illustrated (Figs 23–28). In other Diopsidae genera and species groups, the differences in surstyli are often much smaller, as can, for instance, be seen in *Eurydiopsis* (Feijen 1999: figs 1–9), the sister genus of *Madagopsina*. The differences in hypandrial claspers are also large in *Madagopsina*, as can be noted by comparing these claspers in the new species (Fig. 20) with those of the other five species (Feijen et al. 2018: figs 178–182). For the closely related Syringogastridae, Marshall et al. (2009) referred to the hypandrial claspers as the “large, setulose ventral lobe” of the hypandrial arms. The short, broad, and heavily sclerotised phallus (Fig. 21) in *M.makayensis* Feijen, Feijen & Feijen, sp. nov. is not only unusual for *Madagopsina*, but for the whole Diopsidae family.

### Morphological differences between the two species groups of *Madagopsina*

Due to the description of a new species in the *M.apollo* species group, the list of differences with the *M.apographica* group has to be somewhat revised. A major difference between the two groups, according to Feijen et al. (2018) concerns the anterior central hyaline wing spot. In the *M.apollo* group this spot was located in cells r1 and r2+3, while in the *M.apographica* group this spot only occurred in cell r2+3 and did not extend into cell r1. However, in *M.makayensis* Feijen, Feijen & Feijen, sp. nov. this spot also does not extend into cell r1, so this character can no longer be used to separate the two groups (compare Figs 9–11 with Figs 12–14). The slight difference in pruinescence of the tergites is now also removed from the list of differences. In the *M.apollo* group the tergites were glossy, while in the *M.apographica* group they were slightly pruinose. In *M.makayensis* Feijen, Feijen & Feijen, sp. nov. the tergites are also slightly pruinose, so this character is now also removed from the list of differences. In Table 1, the new list of differences is presented. Compared with Feijen et al. (2018), the range of some ratios is slightly adapted. In the wing pattern another major difference is now introduced: the width of the dark preapical wing band as compared with the wing length and also as compared with the central wing band. Within the *M.apollo* species group, *M.parvapollina* and *M.makayensis* Feijen, Feijen & Feijen, sp. nov. are more closely related to each other than to *M.apollo*. The latter species stands out by its much larger body size, much shorter inner vertical seta and apical seta, much shorter and bulbous apophysis of the surstylus, and its peculiar subanal plate (heart-shaped with bulbous lateral areas).

**Table 1.** Differential character states for the *Madagopsinaapollo* species group and *Madagopsinaapographica* species group.

Character		* Madagopsina *	
		*apollo* species group	*apographica* species group
head	arista	basal half finely microtrichose	almost bare
thorax	scutum length/width	0.80–0.88	0.93–0.95
	pleurotergal spines	laterally directed	posterolaterally directed
wing	width dark preapical band/wing length	0.18–0.20	0.13–0.14
	dark preapical band	as broad as central band and equal in colour	distinctly narrower than central band and darker
femur 1	ratio length/width ♀	3.4–3.5	4.6–6.0
	ratio length/width ♂	3.2–3.7	4.6–6.3
abdomen	shape	club-shaped	slender
	ratio length/broadest width	≤3	~4
	tergite 3	no spots	one pair of lateral spots
	sternite 2 length/width	1.2–1.6	1.9–2.8
	length St1+St2+St3/ width posterior St2	2.8–3.1	4.1–4.8
genitalia	ratio posterior/anterior arm of phallapodeme	1.1–1.4	0.7–0.9

## Conclusions

The division of *Madagopsina* into the *M.apollo* species group and the *M.apographica* species group is consolidated by the inclusion of the morphological data for *M.makayensis* Feijen, Feijen & Feijen, sp. nov. Within the *M.apollo* group, the new species is more closely related to *M.parvapollina*. The division into two species groups and the closer relationship of *M.makayensis* Feijen, Feijen & Feijen, sp. nov. to *M.parvapollina* is fully supported by the geometric morphometrics analysis of wing shape and the analysis of the allometric data with regard to eye span.

## Supplementary Material

XML Treatment for Diopsidae

XML Treatment for Madagopsina

XML Treatment for Madagopsina apographica

XML Treatment for Madagopsina apollo

XML Treatment for Madagopsina freidbergi

XML Treatment for Madagopsina parvapollina

XML Treatment for Madagopsina tschirnhausi

XML Treatment for Madagopsina makayensis
